# Engineering *Trichoderma*-mediated plant defense against bacterial phytopathogens: Micro-and nanobiotechnological strategies

**DOI:** 10.3934/microbiol.2026002

**Published:** 2026-02-11

**Authors:** Zaryab Shafi, Mohammad Shahid, Atul Singh, G. Bhupal Raj, Akhtar Rasool

**Affiliations:** 1 Department of Biosciences, Integral University, Lucknow, Uttar Pradesh-226026, India; 2 Marwadi University Research Center, Department of Agriculture, Faculty of Science, Marwadi University, Rajkot-360003, Gujarat, India; 3 Department of Agriculture, Koneru Lakshmaiah Education Foundation, Green Fields, Vaddeshwaram, A.P.-522302, India; 4 School of Agriculture, SR University, Warangal-506371; 5 Indonesia Research Center for Molecular Chemistry- National Research and Innovation Agency (BRIN), Tangerang, Province of Banten, 15314, Indonesia; 6 Department of Biotechnology, Manav Rachna International Institute of Research and Studies, Faridabad, Haryana, India

**Keywords:** *Trichoderma*, multi-omics, CRISPR, secondary metabolites, nanoparticle biosynthesis, translational bottlenecks

## Abstract

Bacterial phytopathogens such as *Ralstonia*, *Xanthomonas*, and *Pectobacterium* pose a serious threat to global food security, while overuse of chemical pesticides has led to resistance and environmental concerns. *Trichoderma*, traditionally known for antifungal activity, is emerging as a versatile antagonist of bacterial diseases through antibacterial metabolites, immune response, nutrient competition, and rhizosphere modulation. Multi-omics advances have revealed novel biosynthetic gene clusters and host interaction mechanisms, while CRISPR-based genome editing and synthetic biology approaches are enabling the tailored strains with enhanced biocontrol efficiency. Nanotechnology further contributes by facilitating nanoparticle-mediated biosynthesis and controlled-release formulations, improving stability and targeted field delivery. Despite remaining challenges related to field translation, biosafety, and regulation, the integration of omics, genetic engineering, and nanotechnology establishes *Trichoderma* as a next-generation platform for sustainable and precision crop protection.

## 1. Introduction

Bacterial plant pathogens rank among the most serious threats to global agriculture, leading to substantial yield penalties and persistent challenges to food security [1]. In contrast to fungal diseases, bacterial infections are notoriously difficult to control because of their rapid dissemination, limited curative options, and intricate interactions with host plants and soil microbial communities [2]. Major pathogens such as *Ralstonia solanacearum* (bacterial wilt), *Xanthomonas* spp. (bacterial blight and citrus canker), and *Pectobacterium* spp. (soft rot diseases) collectively account for billions of dollars in crop losses each year [3]. These impacts are particularly severe in tropical and subtropical regions, where warm and humid conditions favor bacterial multiplication, disproportionately burdening smallholder farmers through yield reduction, quality deterioration, and rising disease management costs [4].

Surveillance programs and outbreak reports further highlight the evolving and geographically expanding threat posed by bacterial phytopathogens. Molecular monitoring and first-detection studies continue to redefine pathogen distribution patterns; for instance, *Pectobacterium punjabense* was reported for the first time in the Russian Federation in 2024, emphasizing the dynamic nature of soft-rot pathogen spread [5]. Similarly, population-level analyses and surveillance reports indicate increasing diversity and dispersal of the *R*. *solanacearum* species complex, reinforcing its global quarantine relevance [6]. These findings collectively suggest that bacterial diseases, once confined largely to tropical regions, are increasingly emerging in temperate agroecosystems, intensifying economic risks and regulatory challenges.

Conventional management strategies, including copper-based bactericides, antibiotics, and synthetic chemical pesticides, provide only transient disease suppression and are increasingly limited by resistance development, phytotoxic effects, and environmental concerns [7],[8]. The emergence of antibiotic-resistant strains of *Ralstonia* and *Xanthomonas*, together with stricter regulations on antibiotic use in agriculture, underscores the urgency of identifying safer and more sustainable disease management approaches. Although host resistance breeding remains an important strategy, its durability is often compromised by pathogen genetic variability and the instability of resistance traits under field conditions [9]. Collectively, these limitations highlight the pressing need for environmentally compatible and long-lasting biocontrol alternatives.

Among biological control options, *Trichoderma* spp. have gained prominence as versatile and eco-friendly agents, traditionally recognized for their effectiveness against fungal pathogens [10],[11]. Emerging evidence, however, reveals their underappreciated potential in managing bacterial phytopathogens. *Trichoderma* suppresses bacterial diseases through multiple complementary mechanisms, including competition for nutrients and ecological niches, production of antibacterial secondary metabolites such as peptaibols and terpenoids, and induction of host systemic resistance mediated through salicylic acid, jasmonic acid, and ethylene signaling pathways [12],[13]. In addition, robust root colonization and biofilm formation by *Trichoderma* create a protective microbial barrier that restricts pathogen establishment. Synergistic interactions with other beneficial microorganisms, including Pseudomonas spp. and arbuscular mycorrhizal fungi, further amplify disease suppression and enhance plant vigor [14].

Recent advances in multi-omics, synthetic biology, and micro- and nanobiotechnology are reshaping the development of *Trichoderma*-based biocontrol strategies. Integrated omics approaches—spanning genomics, transcriptomics, proteomics, and metabolomics—have enabled the identification of novel biosynthetic gene clusters and bioactive metabolites linked to antibacterial activity [15],[16]. Genome-editing technologies, particularly CRISPR–Cas systems, along with synthetic biology frameworks, now allow the rational design of strains with improved rhizosphere competence and enhanced metabolite production [17]. Furthermore, nanotechnology-driven formulations, such as nano-encapsulation and nano-emulsions, significantly improve the stability, targeted delivery, and field persistence of *Trichoderma* inoculants [18].

Despite these technological advances, several barriers continue to limit large-scale field adoption. Key challenges include biosafety considerations, variable strain performance across diverse agroecological settings, and the scarcity of long-term, multi-location field validations. Socio-economic constraints, including formulation costs, limited farmer awareness, and regulatory complexities, further restrict widespread implementation [19]. Addressing these issues will require coordinated efforts among microbiologists, molecular biologists, agronomists, and policymakers to translate laboratory innovations into affordable, farmer-centric solutions [20].

Therefore, this review presents an integrated synthesis of the emerging role of *Trichoderma* as a biocontrol agent against bacterial phytopathogens. We consolidate current knowledge on molecular mechanisms, omics-driven discoveries, strain engineering strategies, and micro- and nanobiotechnological innovations, while critically discussing existing challenges and future prospects for developing robust, sustainable, and field-ready *Trichoderma*-based disease management systems.

## 2. *Trichoderma* as a biocontrol agent (BCA)

### 2.1. Biology and ecology of Trichoderma

More than 250 species of the fungal genus *Trichoderma* have been identified worldwide, with continuous taxonomic refinement revealing an even greater level of species diversity [21]. These fungi are widely distributed across diverse ecosystems, including agricultural soils, forest litter, and rhizospheres, a success largely attributed to their rapid mycelial growth, metabolic versatility, and strong root-colonizing ability [22]. Molecular phylogenetic investigations have reshaped *Trichoderma* taxonomy, clustering species into well-defined clades associated with distinct ecological roles such as saprophytism, root endophytism, and antagonism toward plant pathogens [23]. Root colonization by *Trichoderma* is an active and chemically mediated process in which fungal hyphae sense and respond to plant-derived root exudates, including sugars, amino acids, and phenolic compounds [24]. Hydrophobins and carbohydrate-binding proteins play a key role in hyphal adhesion, allowing *Trichoderma* to establish a stable, non-destructive association with the outer layers of plant roots [25].

Beyond pathogen suppression, *Trichoderma* species also contribute significantly to plant growth promotion and nutrient acquisition. They enhance phosphate solubilization, mobilize iron through siderophore production, and secrete organic acids that increase nutrient bioavailability in the rhizosphere [26]. Several *Trichoderma* strains synthesize phytohormones such as indole-3-acetic acid, gibberellins, and cytokinins, which stimulate root branching and improve water and nutrient uptake efficiency [27]. Importantly, *Trichoderma* primes plant immune responses by activating salicylic acid-, jasmonic acid-, and ethylene-mediated induced systemic resistance pathways, thereby strengthening the plant's capacity for rapid and robust defense responses upon pathogen challenge [28].

Through its integrated roles in nutrient mobilization, plant growth promotion, and defense induction, *Trichoderma* represents a core component of sustainable crop protection strategies. Its ecological adaptability and functional versatility underpin its expanding application as an effective biocontrol agent against bacterial phytopathogens.

### 2.2. Traditional roles against fungal pathogens

*Trichoderma* are a well-established biocontrol agent of diverse fungal and bacterial pathogens (Table 1), employing four interconnected mechanisms: Mycoparasitism, antibiosis, competition, and ISR.

**Table 1.** Major bacterial phytopathogens, their impacts, and potential role of *Trichoderma*.

Pathogen	Host crops	Symptoms	Economic impact	Current control strategies	Role of *Trichoderma*	References
*R. solanacearum*	Tomato, potato, tobacco, banana	Bacterial wilt (wilting, vascular browning)	Over $1 billion is lost in solanaceous crops per year worldwide	Crop rotation, resistant varieties, soil fumigation	Primes host immunity, creates antimicrobial metabolites, and engages in nutritional competition	[29]
*X. oryzae* pv. *oryzae*	Rice	Bacterial leaf blight (lesions, leaf drying)	Significant yield losses (10–50%) across Africa and Asia	Resistant cultivars, copper sprays, antibiotics (limited)	Induces systemic resistance in rice; modulates rhizosphere microbiota	[30]
*P. carotovorum*	Potato, carrot, cabbage, soft fruits	Soft rot, stem collapse, tissue maceration	Significant losses during storage and transportation after harvest	Sanitation, chemical bactericides, biocontrol (limited)	releases enzymes that break down cell walls and creates volatiles that stop the growth of pathogens	[31]

Mycoparasitism is a defining feature of *Trichoderma* activity, in which the fungus recognizes host fungi through lectin–carbohydrate interactions and subsequently coils around their hyphae [32]. Following physical contact, *Trichoderma* secretes an array of hydrolytic enzymes, including chitinases, β-1,3-glucanases, proteases, and cellulases, which degrade fungal cell walls, leading to pathogen weakening and lysis [33]. Antibiosis further contributes to pathogen suppression through the production of bioactive secondary metabolites such as peptaibols, gliotoxins, viridins, polyketides, and terpenoids. These compounds disrupt fungal membrane integrity, inhibit spore germination, and interfere with key cellular signaling pathways [34]. Notably, metabolite composition varies among *Trichoderma* strains, resulting in strain-specific biocontrol spectra.

Competition represents another critical mechanism underlying *Trichoderma*-mediated disease suppression. Rapid rhizosphere colonization and efficient nutrient acquisition enable *Trichoderma* to outcompete pathogenic fungi for space and carbon-rich niches on the root surface, thereby limiting pathogen establishment and proliferation [35]. In parallel, *Trichoderma* induces systemic resistance in host plants, priming both structural and biochemical defense responses. Induced systemic resistance is associated with elevated activities of defense-related enzymes such as peroxidases and polyphenol oxidases, along with increased accumulation of phenolics and lignin precursors, collectively restricting pathogen ingress. This defense priming operates synergistically with mycoparasitism and antibiosis to enhance overall plant resilience against fungal invasion [36].

The synergistic action of these mechanisms underpins the success of commercial *Trichoderma*-based biocontrol formulations, which are widely used across horticultural crops, cereals, legumes, and plantations. These formulations reduce fungal disease incidence while lowering dependence on chemical fungicides, contributing to sustainable crop protection.

Importantly, the same ecological strategies and bioactive compounds that enable antifungal activity also provide a foundation for antibacterial potential. For example, secreted metabolites such as peptaibols, polyketides, and terpenoids exhibit broad-spectrum antimicrobial effects, while rapid rhizosphere colonization and ISR enhance plant defenses against bacterial pathogens. Therefore, the well-established antifungal mechanisms of *Trichoderma* support and inform its emerging use as a biocontrol agent against bacterial phytopathogens (Table 2).

**Table 2.** Modes of action of *Trichoderma* against bacterial phytopathogens.

Mechanism	Key molecules/enzymes/metabolites	Target in pathogen	Evidence (model system)	Outcome/Effect on Disease	References
Antibacterial metabolites	Peptaibols (alamethicin, trichorzianines), harzianic acid, sorbicillinoids, gliotoxin, polyketides	Membrane disruption, inhibition of respiration and growth	*R. solanacearum*, *X. oryzae*, *P. carotovorum*	Reduced wilt/blight incidence; pathogen growth inhibition	[37],[38]
Siderophore production	Trichosiderin, hydroxamate-type siderophores, ferrichrome-like compounds	Iron sequestration → nutrient limitation, impaired virulence	*Erwinia amylovora*, *P. atrosepticum*	Suppressed soft rot; reduced bacterial colonization	[39],[40]
Lytic enzymes	Chitinases, β-1,3-glucanases, proteases, lipases, DNases	Degradation of biofilms, extracellular polysaccharides, cell envelopes	*X. campestris*, *Clavibacter michiganensis*, *Ralstonia* biofilms	Biofilm disruption; reduced disease severity	[41],[42]
Immune priming	Small secreted proteins (SSPs), hydrophobins, cerato-platanins, VOCs	Activation of SA, JA, and ET signaling; induced PR proteins	Rice–*X. oryzae*, Tomato–*R. solanacearum*, Potato–*Pectobacterium*	Enhanced host resistance, faster defense response	[43],[44]
Microbiome modulation	VOCs (6-pentyl-α-pyrone, isocyanates, terpenes), secondary metabolites	Competitive exclusion of pathogens, shaping rhizosphere microbiota	Tomato, wheat, and potato rhizosphere studies	Increased beneficial microbes; reduced pathogen abundance	[45],[45]
Quorum sensing interference	Degrading enzymes (AHL lactonases, oxidoreductases), secondary metabolites	Disruption of bacterial quorum-sensing signals (AHLs)	*P. carotovorum* (soft rot), *A. tumefaciens*	Inhibited virulence factor expression and biofilm	[47]
Competition for nutrients and niche	Rapid colonization traits, secretion of organic acids, phosphate solubilization	Outcompetes pathogens for space, carbon, and minerals	Soil microcosm studies with *Ralstonia* and *Xanthomonas*	Pathogen suppression in rhizosphere	[48],[49]
ROS-mediated antibacterial action	Hydrogen peroxide, peroxidases, oxidases	Induces oxidative stress in bacterial cells	*Ralstonia* and *Xanthomonas* interactions	Pathogen lysis; restricted spread	[50]
RNA interference & cross-kingdom signaling (emerging)	Extracellular vesicles (EVs) carrying small RNAs	Silencing of bacterial virulence-related genes	Preliminary reports in *Trichoderma*-bacteria interactions	Novel regulatory suppression; research frontier	[51]

### 2.3. Emerging evidence against bacterial pathogens

While *Trichoderma* species have traditionally been investigated for their antifungal properties, growing evidence highlights their pronounced activity against bacterial phytopathogens. *In vitro*, greenhouse, and metabolomics-based studies reveal a broad and multifaceted antibacterial repertoire. For instance, *T*. *longibrachiatum* SMF2 treatments significantly reduce lesion development in rice by inhibiting bacterial proliferation and inducing host defense–related gene expression against *X. oryzae pv. Oryzae* [52]. Similarly, *T. asperellum* suppresses bacterial wilt in tomato caused by *R. solanacearum* through the action of extracellular metabolites that destabilize bacterial membranes while simultaneously activating jasmonic acid- and ethylene-dependent signaling pathways in the host plant [53]. Culture filtrates of *T. atroviride* have also been shown to inhibit *Pseudomonas syringae* pv. tomato, leading to a marked reduction in leaf spot symptoms in *Arabidopsis thaliana* [54]. In addition, *Trichoderma*-derived metabolites interfere with *Agrobacterium tumefaciens*–mediated tumorigenesis, indicating potential applications in the management of crown gall disease [55].

Mechanistically, *Trichoderma* employs antibacterial strategies that partially overlap with antifungal modes of action but are tailored to bacterial physiology. Specific lipopeptides and polyketides destabilize bacterial membranes, resulting in leakage of cellular contents, whereas siderophores such as coprogen and ferricrocin sequester iron and restrict bacterial virulence. Plants treated with *Trichoderma* further exhibit elevated levels of pathogenesis-related proteins, phenolic compounds, and reactive oxygen species, collectively strengthening structural and biochemical defense barriers [56]. Metabolomic investigations continue to uncover novel low-molecular-weight compounds, some bearing structural similarity to known antibiotics, underscoring the largely untapped chemical diversity of the genus. Integrated multi-omics approaches combining genomics, transcriptomics, and metabolomics are now elucidating biosynthetic gene clusters responsible for antibacterial metabolite production, opening new avenues for metabolic engineering and strain optimization [57].

From an ecological perspective, the dual capacity of *Trichoderma* to suppress both fungal and bacterial pathogens position it as a keystone taxon within the rhizosphere. By stabilizing microbial community structure, promoting beneficial symbioses, and reducing pathogen pressure, *Trichoderma* contributes to resilient soil-plant systems [58]. Current applied research increasingly focuses on translating these biological attributes into field-ready solutions, particularly in cropping systems where bacterial diseases severely constrain productivity and chemical control options are diminishing [59]. Collectively, these findings demonstrate that *Trichoderma* functions not merely as an antifungal agent but as a versatile microbial platform capable of suppressing bacterial pathogens, enhancing plant immunity, and supporting sustainable agricultural practices. Realizing its full potential will depend on integrative research frameworks that combine ecological validation with molecular engineering and metabolomic discovery.

## 3. Mechanisms of *Trichoderma* in suppressing bacterial pathogens

**Figure 1.**
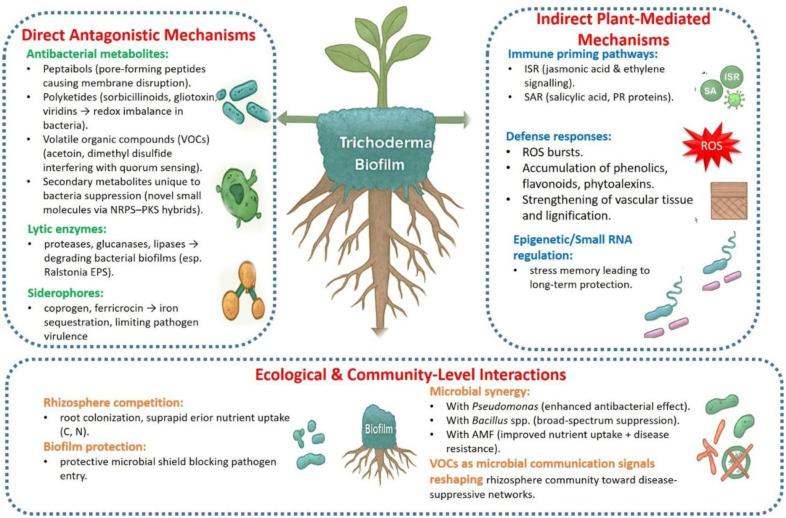
Schematic representation of *Trichoderma* biofilm-mediated suppression of bacterial pathogens through direct antagonism, plant-induced defenses, and rhizosphere ecological interactions.

The mechanism by which *Trichoderma* suppress bacterial phytopathogens is multifactorial, encompassing physiological, ecological, and biochemical dimensions. Antibacterial activity involves diverse metabolites, modulation of host immunity, competitive root colonization, and restructuring the rhizosphere microbiome, extending beyond its well-documented antifungal mechanisms driven by mycoparasitism and cell wall degradation (Figure 1).

Notably, these mechanisms often operate synergistically yet may also compete under certain ecological contexts. For instance, nutrient competition and siderophore-mediated iron sequestration can limit bacterial growth but may reduce the energy available for secondary metabolite biosynthesis. Similarly, strong antibiosis can suppress both pathogens and beneficial microbiota, potentially influencing rhizosphere balance. Conversely, immune priming and moderate competition tend to promote more stable, long-term suppression in complex soil environments. Such trade-offs highlight *Trichoderma*'s adaptive flexibility; its ability to dynamically adjust between competition, antibiosis, and symbiotic modulation depending on plant species, pathogen type, and environmental conditions.

### 3.1. Antibacterial metabolites and lytic enzymes

*Trichoderma* produces a diverse array of structurally heterogeneous metabolites with potent antibacterial properties [60]. Among these, peptaibols—linear peptides containing the non-proteinogenic amino acid 6-aminoisobutyric acid—form voltage-dependent channels in bacterial membranes, disrupting ion gradients and ultimately causing cell lysis [61]. Their agronomic relevance has been demonstrated *in vitro* against *Xanthomonas* and *Pectobacterium* species [62]. Polyketides, including sorbicillinoids, exhibit antibacterial activity against both Gram-positive and Gram-negative phytopathogens by inhibiting cell wall biosynthesis [63]. Gliotoxins and viridins further contribute to bacterial mortality by inducing oxidative stress and disrupting redox homeostasis [64]. Several *Trichoderma* strains harbor hybrid non-ribosomal peptide synthetase-polyketide synthase (NRPS–PKS) gene clusters, expanding the diversity of antibacterial and antifungal compounds and underscoring the genus's largely untapped metabolic potential as a natural antibiotic source [65],[66].

In addition to secondary metabolites, lytic enzymes such as lipases, glucanases, and proteases contribute to bacterial suppression through biofilm degradation, whereas chitinases primarily target fungal pathogens [67]. Because extracellular polysaccharide matrices facilitate bacterial adhesion, colonization, and virulence, enzymatic biofilm disruption exposes pathogens to plant chemical defenses, weakens attachment, and reduces virulence factor expression [68],[69]. Volatile organic compounds (VOCs), including acetoin and dimethyl disulfide, further enhance the antibacterial arsenal of *Trichoderma* by interfering with bacterial signaling and quorum sensing, providing a non-contact mode of action that is particularly effective in complex rhizosphere environments [70],[71]. Collectively, *Trichoderma* deploys a multilayered defense strategy against bacterial phytopathogens that integrates metabolites, enzymes, and volatiles to suppress infection.

### 3.2. Plant immune response modulation

One of the key ecological contributions of *Trichoderma* is immunological priming, which enhances host plant resistance to bacterial invasion. This priming activates both systemic acquired resistance and induced systemic resistance, although these pathways differ in regulatory mechanisms [72]. Induced systemic resistance is primarily regulated by ethylene and jasmonic acid signaling and is associated with the accumulation of pathogenesis-related proteins [73]. *Trichoderma*-treated plants exhibit transcriptional upregulation of genes encoding lipoxygenases, phenylalanine ammonia-lyase, and PR proteins, leading to elevated levels of phytoalexins, phenolic compounds, and reactive oxygen species that create a hostile biochemical environment for bacterial pathogens [74],[75]. Importantly, these responses remain primed rather than constitutively active, enabling rapid defense activation without excessive metabolic cost [76].

Notable examples include *T. asperellum*, which enhances vascular defense in tomato against *R. solanacearum*, thereby reducing wilt incidence [77], and *T. harzianum*, which induces salicylic acid- and jasmonic acid-responsive gene expression in rice to limit *Xanthomonas oryzae* infection [78]. These immune-modulatory effects extend across diverse crops, emphasizing the broad applicability of *Trichoderma*-mediated defense priming [79]. Emerging evidence further suggests that *Trichoderma* may induce long-term immune memory through small RNAs and epigenetic mechanisms, including chromatin remodeling and post-transcriptional gene silencing [80],[81].

### 3.3. Competition for niches and nutrients

Competition for limited resources is a critical determinant of pathogen success in the rhizosphere. *Trichoderma* frequently attains dominance through rapid colonization, efficient nutrient acquisition, and iron sequestration [82]. Iron is essential for bacterial metabolism, quorum sensing, and virulence, making iron limitation a powerful indirect control strategy [83]. *Trichoderma* produces high-affinity siderophores such as ferricrocin and coprogen, which compete effectively with bacterial siderophores and can also influence host iron-associated defense pathways [84]. Spatial exclusion further contributes to pathogen suppression, as *Trichoderma* colonizes root epidermal cells and root hairs, forming physical and chemical barriers that restrict pathogen entry [53].

In addition, localized secretion of antimicrobial metabolites around colonized roots reduces the establishment of soilborne bacterial pathogens such as *Pectobacterium carotovorum*, which rely on wound access for infection [3]. Efficient utilization of carbon- and nitrogen-rich root exudates further limits nutrient availability for competing bacteria [85]. Genomic analyses reveal that many *Trichoderma* strains possess an extensive repertoire of nitrogen transporters and carbohydrate-active enzymes, enhancing their competitive fitness in the rhizosphere [86].

### 3.4. Synergistic interactions with beneficial microbes

Beyond direct antagonism, *Trichoderma* shapes rhizosphere microbial communities in ways that promote plant health and disease suppression [87]. Plant growth-promoting rhizobacteria such as *Bacillus* and *Pseudomonas* spp. frequently co-exist with *Trichoderma*, displaying complementary antibacterial and nematicidal activities [88]. Co-inoculation strategies, for example combining *T. harzianum* with *Pseudomonas fluorescens*, significantly reduce bacterial wilt in tomato while improving crop yield compared with single-microbe applications [89]. Similarly, combined application of *T. asperellum* and *Bacillus subtilis* enhances cucumber resistance to *Pseudomonas syringae* through synergistic production of lipopeptides and hydrolytic enzymes [90].

Tripartite interactions involving *Trichoderma*, arbuscular mycorrhizal fungi, and beneficial bacteria further improve nutrient uptake, root architecture, and systemic resistance, resulting in more resilient plants [91]. These synergistic outcomes arise from coordinated root colonization, complementary metabolite secretion, and modulation of root exudates that selectively favor beneficial microbes [92]. In addition, *Trichoderma*-derived VOCs act as microbial signaling cues that restructure rhizosphere networks, suppressing pathogen proliferation while promoting beneficial taxa [93]. Ecological studies increasingly recognize *Trichoderma* as a stabilizing force within soil microbiomes, capable of preventing pathogen outbreaks under environmental stress [94].

## 4. Development of multi-omics to unravel *Trichoderma*-bacteria-plant interactions

Modern multi-omics technologies have greatly advanced our understanding of complex associations among *Trichoderma*, bacterial phytopathogens, and host plants. These approaches provide a systems-level perspective by integrating genomics, transcriptomics, proteomics, and metabolomics to reveal genetic determinants, regulatory networks, and metabolic fluxes that are inaccessible through traditional microbiological methods. Multi-omics facilitate the rational design of *Trichoderma* strains with enhanced biocontrol potential and enables the systematic discovery of novel secondary metabolites and their biosynthetic pathways, uncovering unknown ecological functions.

### 4.1. Genomics approaches

Genomics-based exploration of *Trichoderma*'s biocontrol repertoire increasingly relies on comparative and functional genomics approaches. High-quality whole-genome sequences are now available for several *Trichoderma* species associated with antibacterial activity, including *T. harzianum*, *T. atroviride*, *T. virens*, and *T. asperellum* [95]. These genomes reveal a marked enrichment of terpene synthases, polyketide synthases, and non-ribosomal peptide synthetase gene families, particularly in strains exhibiting strong antibacterial potential [96]. Many of these biosynthetic gene clusters encode metabolites with both antifungal and antibacterial activities, underscoring the multifunctional nature of *Trichoderma* secondary metabolism [97]. Genome mining has further identified hybrid NRPS–PKS clusters predicted to synthesize novel polyketide–peptide hybrids with antibacterial activity against Gram-negative pathogens such as *R. solanacearum* [98].

Volatile metabolites implicated in quorum-sensing disruption have also been linked to terpene synthase gene clusters not traditionally associated with disease suppression, highlighting previously unrecognized antibacterial pathways [99]. In addition, accessory genes encoding efflux pumps, ATP-binding cassette transporters, siderophore biosynthetic enzymes, and transcriptional regulators of secondary metabolism play important roles in antagonistic interactions, as revealed by genome-scale analyses [100]. Many of these loci exhibit signatures of horizontal gene transfer, reflecting adaptive acquisition of competitive traits in microbially complex soil environments. Pangenome analyses comparing core and accessory genomes among *Trichoderma* strains have further clarified the genetic determinants that distinguish highly effective bacterial antagonists from less potent isolates, thereby guiding targeted strain selection strategies [101]. Collectively, these genomic insights provide a robust framework for informed strain selection and future biocontrol optimization (Table 3).

**Figure 2.**
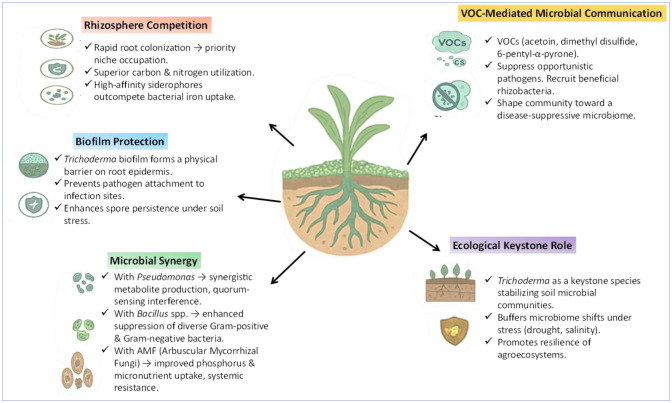
Ecological strategies of *Trichoderma* in the rhizosphere that support pathogen suppression and plant resilience.

**Table 3.** Omics-driven discoveries and engineering advances in *Trichoderma* for antibacterial biocontrol.

Approach	Findings/Targeted gene or cluster	Genes/proteins/metabolites	Tools applied	Improved trait	Model/pathogen tested	Outcome/application relevance
Transcriptomics	Plant defense-related gene upregulation	PR proteins, JA/SA pathway regulators	RNA-seq analysis	Immune priming in host plant	Rice–*X. oryzae*, Tomato–*Ralstonia*	Enhanced resistance to bacterial wilt & blight
Proteomics	Secretome profiling under pathogen challenge	Lytic enzymes, hydrophobins, cerato-platanins	LC-MS/MS	Identification of antibacterial effectors	*T. harzianum* vs *P. carotovorum*	Discovery of candidate proteins for defense
Metabolomics	Novel secondary metabolites with antibacterial activity	Sorbicillinoids, harzianic acid, polyketides	LC-MS, NMR	Novel metabolite identification	Wheat–*X. translucens*	New biocontrol compounds for disease suppression
Genomics/BGC mining	Discovery of cryptic biosynthetic gene clusters (BGCs)	PKS, NRPS, siderophore clusters	Genome sequencing, anti-SMASH	Expanded antibacterial potential	*T. asperellum*, *T. atroviride*	Identification of untapped chemical diversity
CRISPR-Cas editing	Derepression of silent PKS clusters	PKS cluster genes	CRISPR/Cas9 knock-out of repressors	Activation of cryptic metabolites	*T. asperellum* vs *X. oryzae*	Novel antibacterial metabolite production
CRISPR-Cas editing	Overexpression of NRPS genes	Peptaibols	CRISPR-mediated promoter replacement	Increased metabolite secretion	Tomato–*R. solanacearum*	Improved suppression of bacterial wilt
Recombinant DNA	Introduction of bacterial chitinase/glucanase genes	Chitinase, β-1,3-glucanase	Gene transfer via transformation	Enhanced biofilm degradation	*Pectobacterium*spp. biofilms	Reduced biofilm integrity & virulence
Classical mutagenesis	Mutants with elevated metabolite production	Polyketides, lipopeptides	EMS/UV mutagenesis	Higher antibacterial activity	*T. harzianum* vs *R. solanacearum*	Reduced bacterial wilt in tomato
Protoplast fusion	Hybrid strains with broadened antagonism	Siderophores + peptides (from fused strains)	Protoplast fusion	Combined metabolite arsenal	Fusion of *T. asperellum* × *T. viride* vs. *Xanthomonas*	Enhanced suppression of bacterial blight
Synthetic biology	Pathway refactoring for metabolite production	Sorbicillinoid pathway genes	Promoter refactoring, pathway redesign	Higher metabolite yield	Wheat pathogen *X. translucens*	Industrial-scale metabolite production
Heterologous expression	Expression of antibacterial genes from bacteria in *Trichoderma*	Bacteriocin-like peptides	Heterologous expression in fungal host	Expanded antibacterial spectrum	Lab-based antagonism against *Ralstonia*	New activity against bacterial pathogens
Synthetic consortia	Designed microbial communities combining engineered *Trichoderma* + PGPR bacteria	*Trichoderma* metabolites + *Pseudomonas* siderophores	Computational modelling + co-culture design	Multifunctional disease suppression + plant growth	Tomato, potato systems	Broader and more resilient biocontrol

### 4.2. Transcriptomics and proteomics

While genomics provides a static inventory of potential functions, transcriptomics and proteomics uncover the dynamic, context-dependent regulation underlying *Trichoderma*–bacteria–plant interactions. RNA sequencing (RNA-seq) studies have demonstrated that *Trichoderma* extensively reprograms its transcriptome in response to bacterial pathogens and during host colonization [102]. Confrontation assays with *Ralstonia* spp. reveal strong induction of genes encoding hydrolytic enzymes, redox-active proteins, and secondary metabolite biosynthetic machinery [103]. Moreover, plants inoculated with *Trichoderma* and challenged by bacterial pathogens exhibit transcriptional activation of jasmonic acid and salicylic acid signaling pathways, highlighting a multilayered transcriptional dialogue that ultimately shapes disease outcomes [104].

Proteomics complements transcriptomics by identifying the active molecular players involved in these interactions. Mass spectrometry–based secretome analyses have revealed enrichment of small peptides, hydrolytic enzymes, and glycosidases capable of degrading bacterial extracellular matrices [105]. Notably, several small secreted proteins function as signaling molecules that modulate host immune responses, indicating cross-kingdom communication beyond direct antibiosis [106]. Proteomic profiling also uncovers post-translational modifications critical for enzyme activation and secondary metabolite biosynthesis—information not captured by transcript-level analyses alone. Comparative proteomic studies across *Trichoderma* strains further reveal differential abundance of siderophore-binding proteins and detoxification enzymes, providing mechanistic explanations for strain-specific variation in antibacterial efficacy [107]. Collectively, transcriptomic and proteomic datasets establish a mechanistic framework for deciphering the molecular dialogue within the rhizosphere, capturing adaptive fungal responses, plant defense activation, and pathogen counter-strategies.

### 4.3. Metabolomics

Metabolomics bridges the gap between genetic potential and phenotypic expression by directly identifying the chemical arsenal deployed during biocontrol interactions. Advanced analytical platforms such as nuclear magnetic resonance spectroscopy, gas chromatography–mass spectrometry, and liquid chromatography–mass spectrometry have revealed a wide spectrum of antibacterial metabolites produced by *Trichoderma* [108]. Classical compounds including sorbicillinoids, harzianic acid, alamethicin, and trichodermin exhibit strong inhibitory effects on bacterial growth and biofilm formation [109]. Volatile organic compounds such as 6-pentyl-α-pyrone and isonitriles further contribute bacteriostatic activity within plant tissues without compromising host growth [110].

Untargeted metabolomics approaches have uncovered novel small molecules with selective activity against *Xanthomonas* and *Ralstonia* species. Coupling metabolomics with stable isotope labeling has enabled tracing of carbon flux from plant root exudates into antibacterial metabolites, clarifying the metabolic investment of *Trichoderma* during rhizosphere colonization [111]. Integration of metabolomics with genomics allows direct linkage of detected compounds to predicted biosynthetic gene clusters, accelerating natural product discovery [108]. Computational dereplication tools further streamline this process by filtering known compounds and highlighting chemically novel metabolites [112]. Metabolomics also extends to the plant host, where *Trichoderma* inoculation reshapes root and shoot metabolite profiles, enhancing accumulation of phenolics, flavonoids, and phytoalexins that reinforce plant chemical defenses [113],[114]. Together, metabolomics provides a comprehensive view of the chemical cross-talk governing *Trichoderma*–plant–bacteria interactions.

### 4.4. Integrative systems biology approaches

The greatest promise of omics lies in integrative systems biology approaches that synthesize genomic, transcriptomic, proteomic, and metabolomic datasets into holistic models of *Trichoderma*–bacteria–plant interactions. Network-based analyses are particularly powerful, linking biosynthetic gene cluster predictions with transcriptional activation and metabolite production to establish causal relationships from gene to phenotype [115]. For example, integration of RNA-seq and LC–MS metabolomics in *T. asperellum* revealed upregulation of a hybrid non-ribosomal peptide synthetase–polyketide synthase cluster during *Ralstonia* challenge, leading to the production of a novel antibacterial compound [116].

Network visualization platforms enable identification of regulatory hubs controlling biocontrol activity by integrating gene expression, transcription factor dynamics, and metabolite accumulation [117]. On the plant side, combined transcriptomic–metabolomic analyses reveal that *Trichoderma*-induced systemic resistance correlates with coordinated jasmonic acid–responsive gene expression and oxylipin accumulation, which can serve as biomarkers of effective biocontrol [118]. Advanced computational tools enhance these efforts: antiSMASH predicts biosynthetic gene clusters, while STRING and Cytoscape facilitate protein–protein interaction network visualization [119]. Machine learning algorithms applied to multi-omics datasets further enable prediction of key regulatory nodes and candidate targets for strain improvement.

**Figure 3.**
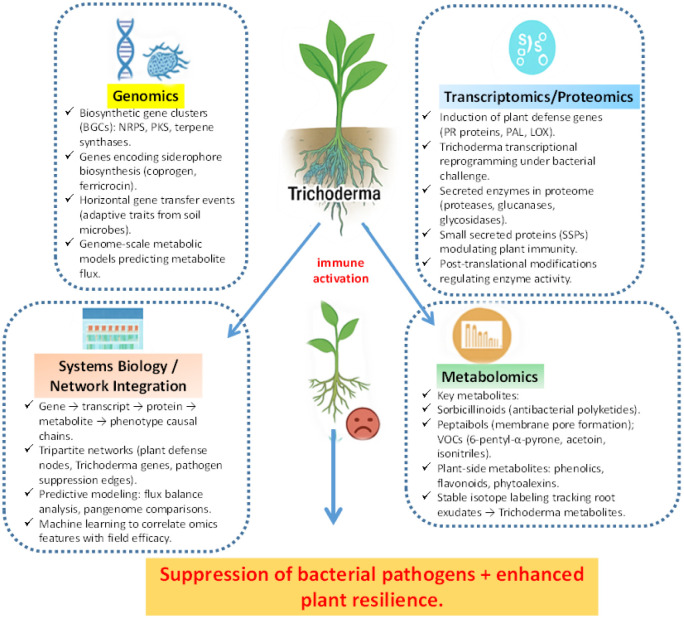
Omics-based insights into *Trichoderma*-mediated suppression of bacterial pathogens and improvement of plant resilience.

Dynamic modeling approaches extend beyond static analyses. Flux balance analysis and genome-scale metabolic models predict how *Trichoderma* reallocates metabolic resources between growth, colonization, and secondary metabolite production under bacterial stress [120]. Importantly, integrative omics reveal the tripartite nature of interactions, emphasizing emergent properties arising from coordinated cross-talk among fungus, plant, and pathogen [121]. These systems-level insights reflect field realities, where disease outcomes depend on complex ecological networks rather than single interactions. The integrated omics framework describing *Trichoderma*-mediated suppression of bacterial pathogens and enhancement of plant resilience is summarized in Figure 3.

### 4.5. Artificial intelligence and computational predictive tools in *Trichoderma*-based biocontrol

Artificial intelligence, machine learning, and deep learning approaches are increasingly applied to analyze complex datasets associated with *Trichoderma*–plant–pathogen systems [122]. These computational tools facilitate prediction of effective *Trichoderma* strains, optimization of biocontrol strategies, and identification of biomarkers governing disease suppression [123]. Machine learning models such as random forests, support vector machines, and neural networks integrate multi-omics datasets to predict biocontrol performance, metabolite production, and strain–host compatibility [124]. Deep learning–based image and spectral analysis platforms support early disease detection and real-time assessment of biocontrol efficacy under field conditions [125].

In parallel, AI-enabled ecological modeling and network-based algorithms advance understanding of rhizosphere microbiome dynamics and support rational design of multi-microbial consortia for durable disease suppression [126]. With expanding data availability, computational tools are poised to play a central role in precision strain selection, formulation refinement, and targeted deployment of *Trichoderma*-based biocontrol agents.

## 5. Engineering *Trichoderma* for enhanced biocontrol

**Figure 4.**
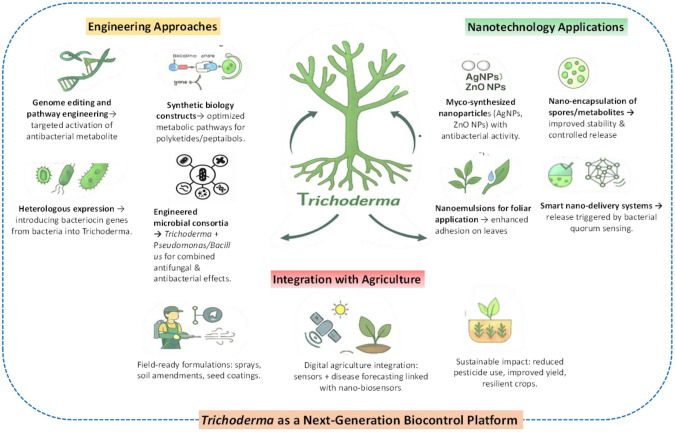
Rhizosphere interactions driven by *Trichoderma* that contribute to plant protection and soil microbial stability.

The recognition of *Trichoderma* as a versatile biocontrol agent has driven efforts to engineer strains with enhanced efficacy against phytopathogenic bacteria. Although natural isolates exhibit diverse antagonistic mechanisms that are valuable for genetic improvement, they also face limitations such as narrow metabolite spectra, inconsistent field performance, and limited persistence in the rhizosphere. Advances from classical mutagenesis to modern CRISPR/Cas-based editing and synthetic biology now offer powerful tools to tailor *Trichoderma* strains for superior suppression of bacterial diseases (Figure 4).

### 5.1. Classical and molecular approaches

Early attempts to improve *Trichoderma* relied mostly on classical mutagenesis and protoplast fusion. Random mutagenesis using chemical agents such as EMS or UV radiation produced variants with increased synthesis of antibacterial metabolites, including polyketides and lipopeptides [127]. Against *R. solanacearum*, mutagenized strains of *T. harzianum* exhibited enhanced inhibition of bacterial wilt due to elevated secretion of diffusible secondary metabolites [128].

Protoplast fusion provides a complementary approach, enabling the combination of desired traits from different species into hybrid strains with broadened antagonistic potential [129]. For instance, fusion between *T. asperellum* and *T. viride* yielded progeny capable of producing siderophores and antibacterial peptides, thereby improving suppression of *Xanthomonas oryzae* [130]. Although outcomes are often unpredictable due to uncontrolled genetic rearrangements, these methods demonstrate the feasibility of creating strains with expanded biochemical repertoires. The subsequent phase of targeted improvement employed recombinant DNA technology, introducing bacterial chitinase and glucanase genes into *Trichoderma* to enhance enzymatic degradation of *Pectobacterium* biofilms [131]. While these proof-of-concept studies showcased the potential for rational strain enhancement, broader application is limited by unstable gene integration, variable expression, and reduced ecological fitness. Nonetheless, these foundational molecular approaches paved the way for the precision era ushered in by CRISPR and synthetic biology.

### 5.2. CRISPR/Cas-based genome editing

The advent of CRISPR/Cas technology has revolutionized *Trichoderma* engineering by enabling precise, efficient, and multiplexed genetic modifications [132]. Unlike random mutagenesis, CRISPR enables targeted activation, silencing, or editing of genes within biosynthetic gene clusters that govern production of metabolites essential for bacterial antagonism [133]. For example, overexpression of non-ribosomal peptide synthetase genes enhanced synthesis of antibacterial peptaibols in tomato-associated *Trichoderma*, resulting in improved suppression of *Ralstonia* infections [134]. Similarly, deletion of transcriptional repressors via CRISPR derepressed cryptic polyketide synthase clusters, revealing novel metabolites active against *Xanthomonas* [135]. These studies highlight CRISPR's potential to unlock hidden chemical diversity within the *Trichoderma* genome and strengthen its antibacterial repertoire.

Beyond metabolite engineering, CRISPR facilitates creation of strains with superior rhizosphere competitiveness and colonization efficiency. Knock-in constructs introducing fluorescent or luminescent reporter genes enable real-time visualization of *Trichoderma*–bacteria–plant interactions and functional screening of engineered strains under greenhouse conditions [136]. The multiplexing capability of CRISPR is particularly advantageous for developing complex traits. By simultaneously editing regulatory genes, membrane transporters, and siderophore biosynthesis pathways, researchers have generated strains exhibiting synergistic improvements in metabolite secretion, persistence, and nutrient competition. Such multifactorial designs hold promise for effective control of multi-host bacterial diseases, including those caused by *Pectobacterium* and *Agrobacterium* [137].

### 5.3. Synthetic biology approach

Synthetic biology extends far beyond single-gene editing, offering a framework to rationally design metabolic pathways or microbial consortia. Pathway refactoring in *Trichoderma* has demonstrated the ability to reorganize biosynthetic gene clusters for more efficient metabolite production [138]. For instance, redesigning the sorbicillinoid biosynthetic pathway significantly enhanced antibacterial metabolite yields, improving suppression of the wheat pathogen *Xanthomonas translucens* [139].

Heterologous expression further expands the antimicrobial repertoire of *Trichoderma* by enabling incorporation of genes from other microorganisms [140]. Expression of bacteriocin-like peptides—typically absent in fungi—broadens antagonistic activity toward bacterial targets. Synthetic promoters and regulatory elements refine metabolic flux, ensuring energy-efficient and context-specific metabolite synthesis. Inducible regulatory systems have been proposed to minimize metabolic burden during normal colonization while activating metabolite production only upon pathogen detection [141], including promoters responsive to bacterial quorum-sensing molecules [142].

At a higher organizational level, synthetic biology enables construction of *Trichoderma*-based microbial consortia. Co-cultivation of engineered *Trichoderma* strains with *Pseudomonas fluorescens* or *Bacillus subtilis* generates multifunctional communities with combined antifungal, antibacterial, and plant growth–promoting traits [143]. Designed consortia offer broader and more durable control against complex bacterial diseases such as wilt and blight, which exploit multiple ecological niches [144].

### 5.4. Risks, biosafety, and regulatory concerns

Regulatory and biosafety considerations significantly influence advancements in *Trichoderma* engineering. Potential ecological impacts remain a major concern, particularly for genetically modified strains with altered metabolite profiles [145]. Risks include accumulation of novel compounds in the food chain, disruption of beneficial microbiota, and horizontal gene transfer to native soil bacteria [146]. Another concern is inhibition of non-target beneficial microorganisms such as nitrogen-fixing endophytes or symbiotic *Rhizobium* species, potentially compromising ecosystem services [147]. Persistence of engineered strains beyond intended cropping cycles may further alter native microbial community dynamics [148].

Risk-assessment frameworks have been established globally to address these concerns. OECD guidelines emphasize case-by-case environmental risk evaluation, while EFSA and EPA frameworks focus on molecular characterization, environmental fate, and non-target effects prior to field release. At the international level, the Cartagena Protocol on Biosafety outlines standards for safe handling and use of genetically modified microorganisms.

To mitigate risks, synthetic biology offers containment strategies such as genetic kill switches, synthetic auxotrophy, and inducible gene circuits activated only under specific environmental cues [149]. CRISPR-based kill switches and synthetic auxotrophy have been successfully demonstrated in bacterial and yeast systems [150]–[152], and similar biosafety circuits are being explored for filamentous fungi, including *Trichoderma*. Conditional expression systems restrict antibacterial metabolite synthesis to pathogen presence, minimizing non-target exposure. Transparent risk communication and long-term monitoring will be essential for regulatory acceptance and public confidence.

## 6. *Trichoderma*-based nanotechnology in biocontrol

### 6.1. Nanoparticle biosynthesis by *Trichoderma*

*Trichoderma* species have emerged as sustainable biofactories for synthesis of metallic and metal-oxide nanoparticles, a process referred to as myco-nanotechnology [153]. Species such as *T. harzianum*, *T. viride*, *T. asperellum*, and *T. atroviride* produce reducing metabolites, extracellular enzymes, and cell-wall-associated proteins capable of transforming inorganic metal salts into stable nanostructures. Silver, zinc oxide, silica, and gold nanoparticles are most extensively studied.

Biosynthesis typically involves bio-reduction of metal ions by fungal metabolites [154], followed by stabilization through protein and polysaccharide capping that prevents aggregation [155]. The resulting nanoparticles are generally uniform, stable, and functionalized with biomolecules that enhance affinity toward bacterial cells (Table 4). Compared with chemical or physical methods, *Trichoderma*-mediated nanoparticle production is eco-friendly, cost-effective, and scalable [156]. Importantly, natural biomolecular coatings improve nanoparticle biocompatibility with plant tissues while minimizing environmental toxicity, supporting sustainable agricultural deployment [157].

**Table 4.** Nanotechnological applications in *Trichoderma*-based biocontrol formulations.

Nanomaterial type	Functional role (delivery, stability, biosynthesis)	Application method	Advantages over conventional formulations	References
Silver nanoparticles (Ag-NPs)	Antibacterial activity; biosynthesized by *Trichoderma* metabolites and enzymes	Foliar spray; soil drench	Strong ROS generation, membrane disruption; enhanced activity vs *Ralstonia*, *Xanthomonas*; eco-friendly biosynthesis	[158],[159]
Zinc oxide nanoparticles (ZnO-NPs)	ROS production; biofilm disruption, nutrient modulation	Soil amendment; seed coating	Effective against *Pectobacterium*; stable under field conditions; plants biocompatible	[160],[161]
Silica nanoparticles (SiO₂-NPs)	Encapsulation of spores/metabolites; controlled release	Root treatment; foliar application	Improved shelf life, UV stability, and sustained release of *Trichoderma* spores	[162],[163]
Polymeric nano-capsules (e.g., alginate, chitosan)	Encapsulation of spores or metabolites; protection against stressors	Soil application; seed coating	Higher spore viability; controlled colonization; protection under drought and heat stress	[164],[165]
Lipid nanocarriers/nano-emulsions	Enhanced dispersal, adhesion, and penetration on plant surfaces	Foliar spray	Better leaf coverage; protection against desiccation; prolonged field persistence	[166],[167]
Gold nanoparticles (Au-NPs)	Biosynthesized by *Trichoderma*; antibacterial and signaling interference	Foliar application	Safe, stable nanoparticles; quorum-sensing inhibition in bacterial pathogens	[168]
Nanogels/nanocomposites	Multifunctional carriers for *Trichoderma* + partner microbes or metabolites	Soil amendment; foliar spray	Co-delivery of beneficial microbes; synergistic protection against bacterial and fungal diseases	[169],[170]

### 6.2. Antibacterial activities of *Trichoderma*-mediated NPs

*Trichoderma*-derived nanoparticles (NPs) exhibit antibacterial activity that extends beyond their nanoscale dimension. Their efficacy arises from the synergistic interaction between the metallic core and fungal biomolecules coating their surface. Multiple mechanisms act concurrently to suppress phytopathogenic bacteria. A primary pathway involves the generation of reactive oxygen species (ROS). Silver (Ag-NPs) and zinc oxide nanoparticles (ZnO-NPs) induce the production of hydrogen peroxide, superoxide anions, and hydroxyl radicals in bacterial cells, leading to oxidative stress that damages proteins, lipids, and DNA, ultimately causing cell death [171],[172]. Another key mechanism is membrane disruption, in which electrostatic interactions between positively charged nanoparticles and negatively charged bacterial cell walls destabilize the lipid bilayer, increase membrane permeability, and result in rapid lysis [173].

Additionally, released metal ions bind to thiol groups of bacterial enzymes, inactivating essential proteins involved in DNA replication, respiration, and cell division [174]. *Trichoderma*-synthesized nanoparticles also degrade extracellular polymeric substances (EPS), dismantling biofilms formed by pathogens such as *R. solanacearum* and *X. campestris*, thereby reducing their virulence and persistence [175]. Experimental studies confirm these mechanisms. For example, Ag-NPs synthesized by *T. asperellum* strongly inhibit *R. solanacearum* (wilt) and *Xanthomonas* spp. (leaf spot), while ZnO-NPs effectively suppress *Pectobacterium carotovorum*, the causal agent of soft rot [176]. Notably, *Trichoderma*-mediated nanoparticles often outperform chemically synthesized counterparts, as the fungal metabolites coating their surfaces enhance nanoparticle stability and broaden antibacterial activity spectra [177].

### 6.3. Nano-formulations for *Trichoderma* delivery

Nanotechnology is not limited to the production of bioactive nanoparticles but also enhances the delivery, stability, and field performance of *Trichoderma*-based formulations. Conventional spore or mycelial preparations are often unstable under extreme temperatures and ultraviolet exposure, with viability declining under fluctuating humidity and storage conditions. These limitations can be mitigated through encapsulation technologies. Lipid nanocarriers and silica nanogels embedding *Trichoderma* spores or metabolites protect the biocontrol agent and enable slow, controlled release into the rhizosphere, thereby improving colonization and pathogen suppression [178],[179]. Similarly, nano-emulsions improve adhesion, spreading, and penetration on plant surfaces, making them effective against foliar bacterial diseases such as blights and leaf spots [180]. These nanotechnology-driven innovations extend shelf life and field stability, with spores in nanoparticle-stabilized formulations remaining viable for several months to over a year [181]. Nano-encapsulated *T. harzianum* has also demonstrated superior root colonization and enhanced protection against bacterial wilt under field conditions compared with conventional formulations [182]. Consequently, nano-enabled delivery systems improve the precision, consistency, and predictability of *Trichoderma*-based biocontrol while reducing application frequency and production costs.

### 6.4. Integration of nanotechnology with omics and engineering

The next generation of *Trichoderma*-based biocontrol is being shaped by the integration of genomics, transcriptomics, metabolomics, and synthetic biology with nanotechnology. One emerging concept involves intelligent nano-delivery systems in which engineered *Trichoderma* strains overproduce key antibacterial metabolites such as peptaibols or polyketides and are embedded in responsive nanocarriers that release bioactive compounds upon detecting pathogen-associated signals, including quorum-sensing molecules, ensuring localized and timely delivery at infection sites [183]. Complementary approaches involve nano-biosensors functionalized with *Trichoderma*-derived enzymes or metabolites for early detection of bacterial phytopathogens in soil and plant tissues, enabling timely and targeted disease management interventions [184].

Omics-guided nano-formulations further refine this strategy by identifying highly bioactive *Trichoderma*-derived compounds, such as sorbicillinoids or alamethicin, which are selectively incorporated into nanocarriers for targeted suppression of pathogens like *Xanthomonas* and *Ralstonia*. Nanotechnology also supports the development of synthetic microbial consortia, where nanostructured carriers co-deliver *Trichoderma* with beneficial microbes such as *Pseudomonas* or *Bacillus*, promoting synergistic interactions and resilient rhizosphere communities [185].

Collectively, these integrative innovations point toward a precision-driven biocontrol paradigm in which *Trichoderma* functions as both a biological agent and a biotechnological platform for sustainable crop protection. However, these strategies remain largely at experimental or proof-of-concept stages, and challenges related to scalability, biosafety, environmental fate, and regulatory compliance must be addressed through long-term interdisciplinary research before widespread agricultural adoption.

Potential ecological and biosafety risks associated with nano-formulations also warrant careful evaluation. Although *Trichoderma*-based nanomaterials are generally considered eco-friendly, excessive accumulation of metallic nanoparticles (e.g., Ag-NPs or ZnO-NPs) in soil may disrupt microbial community structure [186], interfere with nutrient cycling [187], or exert unintended toxicity on beneficial microorganisms, soil fauna, and aquatic ecosystems [188]. Nanoparticle persistence, leaching, and potential bioaccumulation through the food chain further raise environmental and human-health concerns [189]. Therefore, comprehensive risk assessment under realistic field conditions is essential to ensure the safe and sustainable deployment of nano-enabled *Trichoderma* formulations.

### 6.5. Commercial *Trichoderma*-based biocontrol products for managing bacterial plant diseases

Several *Trichoderma*-based microbial biopesticides have been commercialized worldwide and are increasingly integrated into disease-management programs. Products based on *T. harzianum* (e.g., strain T-22), *T. viride*, and *T. asperellum* are widely applied as seed treatments, soil drenches, and foliar sprays in vegetables, cereals, pulses, and horticultural crops [190]. Although most formulations are registered primarily against fungal pathogens, accumulating greenhouse and field evidence indicates that commercial *Trichoderma* products also contribute to the suppression of economically important bacterial diseases, including bacterial wilt, bacterial spot, and soft rot. These effects are mediated through rhizosphere competition, rapid root colonization, antibiosis, microbiome modulation, and induction of systemic resistance, resulting in reduced disease incidence and improved plant performance [191]. Table 5 summarizes representative commercial formulations, their active strains, application modes, and reported activity against major bacterial pathogens.

**Table 5.** Commercial *Trichoderma*-based products used in plant disease management.

Commercial product	Active ingredient (species/strain)	Formulation/use	Application	Reported bacterial relevance (direct/indirect)	References
RootShield®/PlantShield®/Trianum® (commercial strain T-22)	*T. harzianum* (currently often *T. afroharzianum*) strain T-22	WP/WG	Seed/soil/transplant drench	Strong ISR + microbiome effects; used in disease complexes	[192]
Trianum® (T-22)	*T. harzianum* T-22	WG/granule	Soil/substrate	Defense priming and antioxidant/phytohormone regulation (supports bacterial disease suppression indirectly)	[193]
TRICHODEX®	*T. harzianum* T-39	Biofungicide	Foliar/soil	Systemic defense elicitation may suppress bacterial diseases indirectly	[194]
VINTEC®	*T. atroviride* SC1	WG	Wound protection	Alters grapevine defense transcriptome; supports protection in microbial disease complexes	[195]
VINTEC®	*T. atroviride* SC1	WG	Vineyard wound	Demonstrated biological control performance under field disease pressure (supports ID management)	[196]
Remedier®	*T. asperellum* ICC012 + *T. gamsii* ICC080	WP	Soil/wounds	Improves plant protection responses; supports microbial community-mediated disease suppression	[197]
Bio-Tam®	*T. asperellum* ICC012 + *T. gamsii* ICC080	Biofungicide	Soil/root	Strain-based evidence in crop protection; supports indirect suppression	[198]
ASPERELLO® T34/T34 Biocontrol®	*T. asperellum* T34	WP	Soil/root	Enhances plant defense; supports suppression of bacterial/complex diseases indirectly	[199]
Generic *T. harzianum* WP/SC	*T. harzianum* (various strains)	WP/SC	Soil/seed	Suppression of bacterial blight context via induced defense (evidence from rice bacterial blight model)	[200]
*Trichoderma* spp. commercial biofungicides (global)	*Trichoderma* spp.	WP/WG/SC	Seed/soil	Broad bacterial disease reduction through ISR, antioxidants, and phytohormones	[201]

## 7. Challenges and future perspectives

The growing body of research highlights *Trichoderma* as a promising biocontrol agent (BCA) against bacterial phytopathogens, supported by biological efficacy, technological innovations, and regulatory frameworks that make large-scale deployment feasible. However, significant knowledge gaps remain regarding its antibacterial mechanisms. Although several metabolites, including peptaibols, sorbicillinoids, and volatile organic compounds (VOCs), have been identified, their chemical diversity, regulatory networks, and specific bacterial targets remain poorly understood, particularly under field conditions where plant immune responses mediated by induced systemic resistance (ISR) and systemic acquired resistance (SAR) are highly variable [202],[203].

Translational challenges persist, as *Trichoderma* efficacy—often robust under controlled laboratory and greenhouse conditions—is frequently reduced in the field due to complex soil microbiomes, fluctuating environmental stresses, and pathogen diversity. Inconsistent rhizosphere colonization and variable metabolite production further limit reliability and reproducibility of disease suppression [204]–[206]. Moreover, multi-omics approaches continue to generate valuable mechanistic insights, underscoring the need for predictive frameworks that integrate genomics, ecology, and machine learning (ML) to forecast field-level performance and stability [207].

Deployment is further complicated by regulatory and biosafety constraints. CRISPR-edited strains and nanoparticle-based formulations face uneven and often stringent regulatory policies across global markets that prioritize biosafety but substantially delay commercialization [208],[209]. Environmental concerns also persist, as the release of genetically engineered strains or excessive nanoparticle accumulation may alter soil microbial communities or impact non-target organisms, highlighting the importance of long-term ecological risk assessments [210]–[212].

Beyond scientific and regulatory challenges, socio-economic barriers continue to limit large-scale adoption of *Trichoderma*-based BCAs. High formulation costs, limited market availability, and low awareness among smallholder farmers constrain uptake, particularly in developing agricultural systems. Weak extension services, inconsistent product quality, and insufficient policy incentives further reduce farmer confidence in bio-based inputs. Addressing these issues through farmer education, financial support mechanisms, and standardized certification frameworks will be essential to promote broader adoption and trust in *Trichoderma*-based technologies.

Despite these challenges, future prospects remain highly encouraging. Advances in strain engineering and omics-driven discovery pipelines are accelerating the identification of novel antibacterial compounds, while nanotechnology is enabling the development of stable and tailored formulations suited to diverse agroecosystems. Integration of digital agriculture tools within integrated pest management (IPM) frameworks can further enhance precision and reliability. For example, remote sensing and ML-based disease forecasting systems can be used to identify pathogen hotspots and optimize the timing of *Trichoderma* applications in crops such as baby lettuce [213]. Scaling up adoption will require strong public–private partnerships, farmer capacity building, and supportive regulatory frameworks [214],[215]. Through coordinated innovation and interdisciplinary collaboration, *Trichoderma*-based solutions can transition from experimental success to mainstream biocontrol technologies, contributing to sustainable crop productivity and long-term ecological resilience.

## 8. Conclusion

Bacteria phytopathogens cause major agricultural losses worldwide, underscoring the need for sustainable alternatives to chemical pesticides. *Trichoderma*, traditionally recognized for its strong antagonistic activity against plant pathogens, has emerged as a potent biological control agent (BCA). They produce diverse antibacterial metabolites, modulate plant defense responses, compete for nutrients, and reshape the rhizosphere microbiomes, making them a versatile BCA. Advances in nanotechnology, genome editing, and synthetic biology are accelerating the development of optimized *Trichoderma* strains and targeted delivery systems, while multi-omics studies continue to reveal the molecular complexity of plant–microbe interactions. Moving forward, an interdisciplinary framework integrating microbiology, plant pathology, molecular biology, bioinformatics, agronomy, and regulatory science is vital for translating these advances into field-ready solutions. Active collaboration among researchers, industry partners, extension services, and policymakers will ensure the safe, scalable, and effective deployment of *Trichoderma*-based technologies. Thus, harnessing *Trichoderma*'s potential can enhance crop resilience, reduce reliance on chemical bactericides, and significantly contribute to sustainable agriculture and global food security.

## Use of AI tools declaration

The authors declare they have not used Artificial Intelligence (AI) tools in the creation of this article.
